# Validity of genus *Perostrongylus* Schlegel, 1934 with new data on *Perostrongylus falciformis* (Schlegel, 1933) in European badgers, *Meles meles* (Linnaeus, 1758): distribution, life-cycle and pathology

**DOI:** 10.1186/s13071-018-3124-x

**Published:** 2018-10-30

**Authors:** Georgiana Deak, Andrei Daniel Mihalca, Joerg Hirzmann, Vito Colella, Flaviu Alexandru Tăbăran, Maria Alfonsa Cavalera, Florinel Gheorghe Brudașcă, Christian Bauer, Angela Monica Ionică, Amer Alić, Domenico Otranto, Călin Mircea Gherman

**Affiliations:** 1 Department of Parasitology and Parasitic Diseases, University of Agricultural Sciences and Veterinary Medicine Cluj-Napoca, Cluj-Napoca, Romania; 2 Institute of Parasitology, Justus Liebig University Giessen, Giesen, Germany; 3 Dipartimento di Medicina Veterinaria, Università degli Studi di Bari, Str. prov. per Casamassima km 3, 70010 Valenzano, Bari Italy; 4 Department of Pathology, University of Agricultural Sciences and Veterinary Medicine Cluj-Napoca, Cluj-Napoca, Romania; 5 Department of Game and Wildlife Diseases, University of Agricultural Sciences and Veterinary Medicine Cluj-Napoca, Cluj-Napoca, Romania; 6 Department of Pathology, Faculty of Veterinary Medicine, University of Sarajevo, Zmaja od Bosne 90, 71000 Sarajevo, Bosnia and Herzegovina

**Keywords:** *Perostrongylus*, *Aelurostrongylus*, Metastrongyloidea, European badgers, mustelids

## Abstract

**Background:**

A century of debates on the taxonomy of members of the Metastrongyloidea Molin, 1861 led to many reclassifications. Considering the inconstant genus assignation and lack of genetic data, the main aim of this study was to support the validity of the genus *Perostrongylus* Schlegel, 1934, previously considered a synonym of *Aelurostrongylus* Cameron, 1927, based on new molecular phylogenetic data and to understand its evolutionary relationships with other metastrongyloid nematodes.

**Results:**

Specimens of lungworm collected from European badgers in Germany, Romania and Bosnia and Herzegovina were morphologically and molecularly (rDNA, *cox*1) characterized. From a phylogenetic standpoint, *Perostrongylus* is grouped with high support together with the genera *Filaroides* van Beneden, 1858 and *Parafilaroides* Dougherty, 1946 and includes probably two species: *Perostrongylus falciformis* (Schlegel, 1933), a parasite of *Meles meles* in Europe and *P. pridhami* (Anderson, 1962), a parasite of *Neovison vison* in North America. *Perostrongylus* and *Aelurostrongylus* are assigned to different clades. *Aelurostrongylus* becomes a monotypic genus, with the only species *Aelurostrongylus abstrusus* (Railliet, 1898). In addition, we provide morphological and morphometric data for the first-stage (L1), second-stage (L2), and third-stage (L3) larvae of *P. falciformis* and describe their development in experimentally infected *Cornu aspersum* snails. The pathological and histopathological lesions in lungs of infected European badgers are also described. This is the first record of *P. falciformis* in Romania.

**Conclusions:**

Molecular phylogenetic and morphological data support the validity of the genus *Perostrongylus*, most probably with two species, *P. falciformis* in European badgers and *P. pridhami* in minks in North America. The two genera clearly belong to two different clades: *Perostrongylus* is grouped together with the genera *Filaroides* and *Parafilaroides* (both in the family Filaroididae Schulz, 1951), whereas *Aelurostrongylus* belongs to a clade with no sister groups.

## Background

A century of debates on the taxonomy of members of the superfamily Metastrongyloidea Lane, 1917 led to many reclassifications of these nematodes within a variety of families, subfamilies, genera and species [1, 2]. The genus *Aelurostrongylus* Cameron, 1927 was erected to accommodate *Aelurostrongylus abstrusus* (Railliet, 1898) [3] which was originally described by Mueller in 1890 [4] as *Strongylus pusillus* and renamed to *Strongylus abstrusus* by Railliet [5], because *S. pusillus* was preoccupied [6]. Later, the genus *Protostrongylus* Kamensky, 1905 was erected [7] and *Strongylus abstrusus* transferred to this new genus (as *Protostrongylus pusillus*), containing two other species, i.e. *Protostrongylus rufescens* (Leuckart, 1865), the type-species, and *Protostrongylus commutatus* (Diesing, 1851) [as *Protostrongylus terminalis* (Passerini, 1884)]. Interestingly, Kamensky used the early specific name as originally named by Mueller in 1890.

The definition of *Aelurostrongylus* as a new genus [3] was based mainly on the absence of cuticular bursal supports and the absence of “supporting fingers” of the spicular sheaths. Cameron [3] included only the type-species *A. abstrusus* in the newly erected genus.

*Perostrongylus falciformis* (Schlegel, 1933) was described from European badgers, *Meles meles* in Germany as *Strongylus falciformis* Schlegel, 1933 [8] due to the sickle-shaped spicules and one year later, transferred to the genus *Filaroides* van Beneden, 1858 as *Filaroides falciformis* [9]. In the same year, Schlegel [10] erected a new genus, *Perostrongylus* Schlegel, 1934 in order to include this nematode. The main characteristics to support the new genus were the reduced, truncated copulatory bursa of the males, and the presence of larvated eggs in the uterus of the females [10]. A few years later, Wetzel [11] suggested that *P. falciformis* should be transferred to the genus *Aelurostrongylus*, considering the genus *Perostrongylus* as a synonym, based on the morphology of the bursa and gubernaculum in males. In two subsequent reviews on this group of nematodes [12, 13], the genus *Perostrongylus* is listed as a synonym of *Aelurostrongylus*, based on Wetzel’s suggestions, without further comments. In his taxonomic review, Dougherty [12] listed four species in the genus *Aelurostrongylus*: *Aelurostrongylus abstrusus* (Railliet, 1898); *Aelurostrongylus brauni* (von Linstow, 1897); *Aelurostrongylus. falciformis* (Schlegel, 1933); and *Aelurostrongylus fengi* (Hsü, 1935). The latter has been initially described as the type-species of *Pulmostrongylus* Hsü, 1935 [14], but Dougherty [12] considered this genus as a synonym of *Aelurostrongylus* while Anderson [15] and Lesage [16] as a subgenus of *Protostrongylus*. However, Seneviratna [13] as well as the authors of later keys and reviews [17, 18] maintained the validity of *Pulmostrongylus*. The generic allocation of *A. brauni* was also questioned [19]. Asakawa et al. [20] redescribed this species and assigned it to the newly established genus *Viverrostrongylus* Asakawa, 1986.

A new species belonging to genus *Aelurostrongylus*, *A. pridhami* was described by Anderson [21] in *Neovison vison* from Canada. Previously, the species was erroneously identified as *A. falciformis* [22]. The same author [19] highlighted that some species of the genus *Aelurostrongylus* (*A. abstrusus* and *A. fengi*) are oviparous, while others (*A. falciformis* and *A. pridhami*) are ovoviviparous. Moreover, *A. falciformis* and *A. pridhami* differ from *A. abstrusus* with regard to the morphology of the bursa and spicules. Based on these differences, Anderson [19] suggested that the genus *Perostrongylus* should be reinstated, but later placed it as a subgenus of *Aelurostrongylus* [15, 17]. Subsequent publications continued to use the genus name *Aelurostrongylus* (synonymy of the genus [23]; *A. falciformis* [24–29], *A. pridhami* [27, 30–33]), while others accepted and used *Perostrongylus* (*P. pridhami* [34–42], *P. falciformis* [37, 43]). It is evident, that most European studies maintained the validity of *Aelurostrongylus* while the American studies predominantly used *Perostrongylus*. However, based on the data from Anderson [19], the two species are congeneric, sharing the same features.

The life-cycles of both species of *Perostrongylus* have been described in detail. Wetzel [11] studied the development of *P. falciformis* in several snail and slug species, and described the L1-L3 larval stages, but illustrated only L1 and L3. He also established the prepatent period in experimentally infected European badgers. The development of *P. pridhami* was described in slugs as well as in various terrestrial and aquatic snails, with detailed description and illustrations for L1-L3 larval stages [21]. The development of *P. pridhami* in American minks has also been described in detail [21, 37]. Anderson [21] demonstrated the infectivity to minks of L3 of *P. pridhami* from paratenic hosts (rodents, birds, amphibians and fish).

Similarly to most metastrongyloid nematodes, *P. falciformis* has an indirect life-cycle. Females are ovoviviparous and deposit in the alveoli thin-shelled eggs with larvae which subsequently hatch. Larvae and mature adult parasites are found in the alveoli, alveolar ducts and terminal bronchioles. L1 are coughed-up, swallowed and shed through the faeces. Prepatency in experimental infections was shown to be 18–19 days. Larvae enter different species of land snails and moult twice to infective L3. European badgers become infected by eating snails or paratenic hosts. The development of L3 to adults in European badgers is not known [11]. Symptoms of infected European badgers vary from less severe to death, depending on lungworm infection rate and secondary bacterial infections. The associated lung lesions and different stages of verminous pneumonia in European badgers with minor to massive lungworm infections were lobular bronchopneumonia, diffuse bronchitis and widespread emphysema [8, 10]. In minor infections, European badgers in good nutritional status often completely recovered after coughing out the worms, leading to the regression of inflammatory process and encapsulation and calcification of degraded worms and eggs in the form of small, cheesy calcareous nodules in the subpleura and parenchyma of lung lobes. The lungworm invasion affected predominantly young animals [8, 10, 11, 19].

Considering the rather inconstant genus assignation and lack of molecular data, the main aim of this study was to provide information to support the validity of the genus *Perostrongylus* based on new molecular phylogenetic data and to understand its evolutionary relationships with other lungworms of carnivores. In addition, morphological details of the larval stages of *P. falciformis*, as well as a detailed pathological and histopathological description of the lesions in European badgers are provided.

## Methods

### Sample collection

Thirty-two adult European badgers were collected from different localities in Europe (Table 1) and carcasses were examined by necropsy by removing the entire respiratory tract. The trachea, bronchi, and the bronchioles were longitudinally dissected and carefully examined under a stereomicroscope for the presence of parasites. Nematodes found encapsulated in small nodules on the surface of the lungs were collected, washed in saline solution and preserved in formaldehyde for morphological identification. Midbody fragments of nematodes were stored in 70% ethanol for DNA extraction and molecular identification.

**Table 1:** The examined samples and number of European badgers infected with *P. falciformis*

Country	No. of European badgers examined	Positive	Prevalence (%)
Romania	27	9	33.3
Bosnia and Herzegovina	1	1	nd
Germany	4	3	75

*Abbreviation*: *nd* not determined

### Morphological analysis

Eight males and three females collected from two European badgers in Romania (CJ005077 from Hărman, Brașov County and CJ005086, from Charlottenburg, Timiș County) were examined as temporary mounts in lactophenol. Five morphometric features in males (body length, body width, oesophagus length, length of spicules, length of gubernaculum) and six morphometric features in females (body length, body width, oesophagus length, distance between the vulva and the caudal end, distance between the anus and the caudal end, distance between the vulva and anus) were evaluated. Furthermore, 50 L1 larvae collected from the lungs of the same European badgers were also measured (length and maximum width). Measurements were taken using an Olympus BX61 microscope, DP72 digital camera and the Cell^F imaging software (Olympus Corporation, Tokyo, Japan). One male was available for measurements in Germany. No adult specimens were available for measurement from the European badger collected in Bosnia and Herzegovina. Additionally, larvae collected from the experimentally infected snails were also examined.

### Experimental life-cycle of *P. falciformis* in *Cornu aspersum*

First-stage larvae of *P. falciformis* were recovered from the lungs of a naturally infected European badger (CJ005086), hunted in Charlottenburg, Timiș County, Romania (45.975825°N, 21.518763°E) by the Baermann method [44]. The resulted solution was collected into two 50 ml Falcon tubes, centrifuged at 600× *g* for 3 min and the sediment examined under light microscopy. Larvae obtained were morphologically and molecularly identified as *P. falciformis* (based on sequence identity with morphologically confirmed adults). Single infective doses of 200 L1 each were collected and used for the infection of snails. *Cornu aspersum* snails not exposed to any nematodes of vertebrates were purchased from a commercial provider from Puglia, Italy. The snails were kept in a plastic box, covered with a fine mesh, in a temperature-controlled room (21 ± 2 °C) and fed lettuce every second day. Water was provided *ad libitum*. Moreover, the boxes were humidified twice a day. To exclude the presence of any previous parasitic infections, a subset of 10 snails were artificially digested and microscopically examined, one day before the infection. The experimental infection took place in the Unit of Parasitology of the Department of Veterinary Medicine of the University of Bari, Italy. *Cornu aspersum* snails (*n* = 30) were deprived of food 24 h before the infection and then placed individually into infection chambers, composed of six circular cell culture wells (Corning; CellBIND; Sigma-Aldrich, St. Louis, Missouri, USA). Each well contained a potato slice (0.3–0.4 mm thick, obtained with a circular puncher) with the infective dose on the surface. The infection chambers were covered with a wet gauze cloth and secured with rubber bands. The snails were maintained in the infection chamber for 24 h and then released in the rearing box.

Larval development of *P. falciformis* was assessed by artificial digestion [45] of five randomly selected snails at 3 (T1), 6 (T2), 10 (T3), 15 (T4), 20 (T5) and 30 (T6) days post-infection (dpi). At each dpi, the suspension obtained from the gastropod digestion was microscopically examined and, when present, larvae were isolated and preserved in 70% ethanol. Larvae were then cleared and examined as temporary mounts in glycerol and digital images and measurements were taken using Leica LAS® AF 4.1 software.

### Molecular analysis

The specimens used for molecular analysis are shown in Table 2. DNA was isolated from one male, one female fragment and three pools of L1 collected separately from three infected European badgers in Germany, using the DNeasy Blood & Tissue Kit (Qiagen, Hilden, Germany) according to the manufacturer’s protocol. The ribosomal DNA (rDNA) region including partial *18S* rRNA gene, internal transcribed spacer 1 (ITS1), *5.8S* rRNA gene, internal transcribed spacer 2 (ITS2) and partial *28S* rRNA gene and a partial sequence of the mitochondrial cytochrome *c* oxidase subunit 1 gene (*cox*1) were all amplified using nematode-specific primers. For the rDNA region we used combinations of the forward primers N18SF1, NF1 and NC1 and the reverse primers D3B, NC2 and NC5BR [44–49]. For the *cox*1 sequence we used the primers MetCOI-F1 and JB4.5 [50–52]. PCR was performed with HOT FIREPol® Blend Master Mix (Solis BioDyne, Tartu, Estonia), 200 nM final concentration of forward and reverse primers each and 100 ng of nematode DNA in a 50 μl reaction volume. PCR cycling conditions were as follows: 15 min activation/initial denaturation at 95 °C, 35 cycles of 20 s denaturation at 95 °C, 30 s annealing at 54 °C and 2 min extension at 72 °C, followed by a 5 min 72 °C final elongation. Amplicons were analysed on 1.5% agarose gels, gel-purified, cloned into pDrive vector (Qiagen, Hilden, Germany) and sequenced by an external service provider (LGC Genomics, Berlin, Germany). Sequence chromatograms were checked manually and complete sequences, assembled from overlapping amplicons, were submitted to the GenBank database under the accession numbers KY365435-KY365437.

**Table 2:** Samples used for molecular analysis

Sample type	Sample code	Locality of origin	Country	Target gene	Primers	GenBank ID
Adult nematode (f)	CJ005077	Hărman	Romania	ITS2	NC1/NC2^a^	MG733142
Adult nematode (f)	CJ005086	Charlottenburg	Romania			
Adult nematode (f)	CJ005077	Hărman	Romania	*cox*1	LCO/HCO^b^	MG736730
Adult nematode (f)	CJ005086	Charlottenburg	Romania			
Adult nematode (m) + L1 pools	DE-FD-Mm3	Fulda	Germany	rDNA	NC18SF1/D3B	KY365435
Adult nematode (m) + L1 pools	DE-FD-Mm3	Fulda	Germany	*cox*1	MetCOIf1/JB4.5	KY365437
L1	OP137/17	Semizovac	Bosnia and Herzegovina	ITS2	NC1/NC^a^	MG910460
L1-L3 from experimentally infected *Cornu aspersum*				ITS2	NC1/NC2^a^	MG733142

^a^As in [47]

^b^As in [51]

Genomic DNA was extracted from two adult nematodes from Romania, using a commercial kit (Isolate II Genomic DNA Kit, Bioline, UK), according to the manufacturer’s instructions. For each nematode, PCR amplification of a ∼700 bp fragment of the *cox*1 gene and of the internal transcribed spacer 2 (ITS2, ∼500 bp) of the rRNA gene were performed, using primers and protocols available in literature [47, 51]. The amplicons were purified using a commercial kit (Isolate II PCR and Gel Kit, Bioline, UK) and sequenced using an external service (performed by Macrogen Europe, The Netherlands).

Genomic DNA from L1 collected from one European badger from Bosnia and Herzegovina was extracted using a commercial kit (DNeasy Blood & Tissue Kit, Qiagen, GmbH, Hilden, Germany), in accordance with the manufacturer’s instructions, and a partial fragment of the ribosomal internal transcribed spacer 2 (ITS2) was amplified as previously described [47]. Amplicons were purified and sequenced in both directions using the same primers as for PCR, employing the Taq Dye Deoxy Terminator Cycle Sequencing Kit (v.2, Applied Biosystems) in an automated sequencer (ABI-PRISM 377). Sequences were aligned using the Geneious R9 software package (http://www.geneious.com) and compared (BLASTn) with those available in the GenBank database (http:/ blast.ncbi.nlm.nih.gov/Blast.cgi).

Additionally, the DNA was isolated from three specimens of each larval stage collected from experimentally infected snails, following the same protocol used for nematodes from Bosnia and Herzegovina.

### Phylogenetic analysis

For molecular phylogenetic analyses datasets of sequences obtained from BLAST searches of the NCBI nucleotide (nt/nr) database using complete and partial *Perostrongylus* sequences were trimmed to homologous ends and realigned using the multiple sequence alignment program MAFFT 7 [53] with the L-INS-i method for the *28S* D2-D3 and *cox*1 sequence data sets and the structure-aided Q-INS-i method for the ITS2 sequence data set. Phylogenetic trees were constructed using Bayesian analysis (MrBayes 3.2) (10,000 tree generations, sampling each 10, discarding first 250 trees) and TreeDyn for tree drawing at the phylogeny.fr platform [54]. The *28S* D2-D3 data set included 25 taxa and sequences homologous to nucleotides (nt) 3053–3968 of the *P. falciformis* sequence (KY365435). The ITS2 data set included 26 taxa and sequences homologous to nt 2513–2983 (including 15 nt of the flanking *5.8S* rRNA gene and *28S* rRNA gene). The *cox*1 data set included 15 taxa and sequences homologous to nt 200–650 of KY365437.

### Histological analysis

Pieces of lung tissues originating from two fresh (unfrozen) European badgers collected in Romania (CJ005077 from Hărman, Brașov County and CJ005086, from Charlottenburg, Timiș County) containing nodules with the nematodes were fixed in 10% phosphate-buffered formalin for 24 h, routinely processed, embedded in paraffin wax, cut into 4 μm sections, and stained with haematoxylin and eosin (H&E).

## Results

Out of the 32 examined European badgers, *P. falciformis* was found in 13 animals from all countries (Table 1).

### Morphological description of the adults

**Fig. 1:**
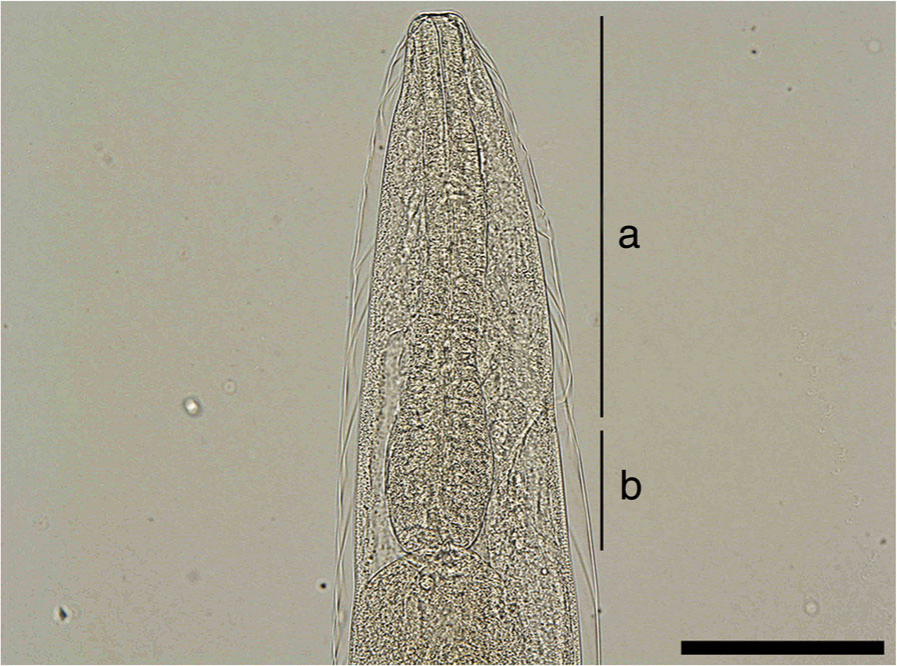
Anterior end of *Perostrongylus falciformis.* **a** Cylindrical part of the oesophagus. **b** Bulbous region of the oesophagus. *Scale-bar*: 100 μm

Adult worms show a pronounced sexual dimorphism, the females being larger than the males. Both sexes have a cylindrical body, uniformly coloured, elongated, thread-like, very thin and extremely coiled inside nodules, making the removal of intact specimens difficult. The cuticle at the anterior end is smooth and the mouth opening is placed terminally. The buccal cavity is small, rudimentary, and opened into a clavate oesophagus which is composed of a cylindrical part in the anterior two-thirds of its length and a posterior bulbous region (Fig. 1).

**Fig. 2:**
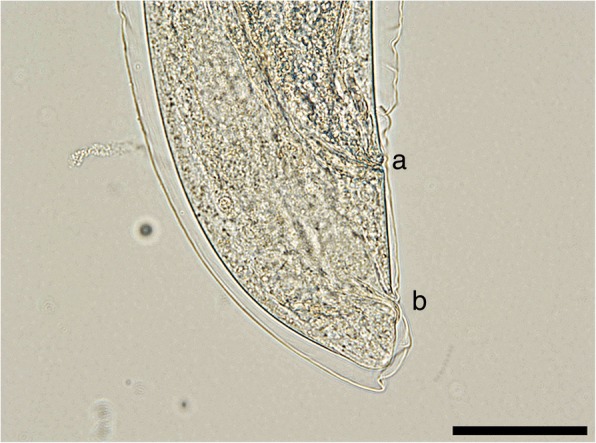
Posterior end of female *P. falciformis*. **a** Vulva. **b** Anus. *Scale-bar*: 50 μm

**Fig. 3:**
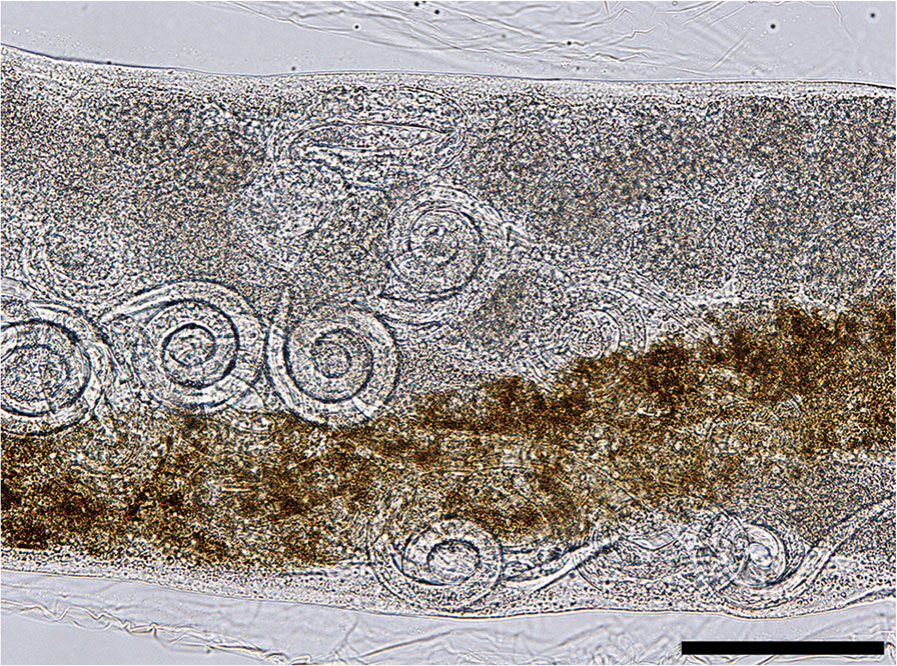
Larvated eggs are clearly visible inside the uterus. *Scale-bar*: 100 μm

**Fig. 4:**
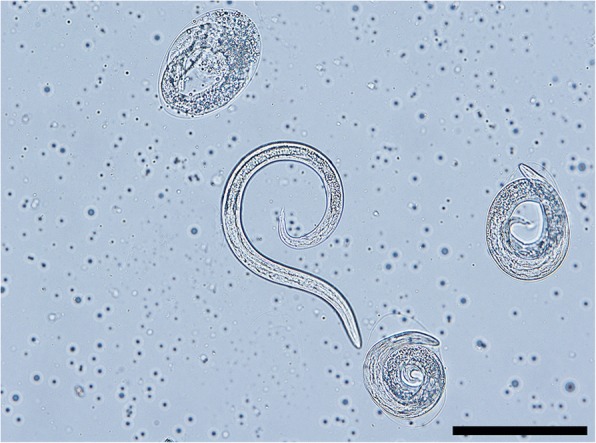
Free L1 larva (center) and eggs with larvae of *P. falciformis. Scale-bar*: 100 μm

The posterior end of females is slightly curved with the vulvar and anal openings on the lower curvature (Fig. 2). Morphometric data are shown in Table 3. In the uterus, larvated eggs are clearly visible (Fig. 3), demonstrating the ovoviviparity (Fig. 4).

**Table 3:** Comparative morphometric data for *Perostrongylus falciformis* obtained in the present study. Measurements are in micrometres unless indicated otherwise

Country	Romania	Germany^a^
Male	(*n* = 8)	(*n* = 1)
Body length (mm)	11.9–23.7	26.0
Body width	136–328	160
Cuticle thickness at mid-body	4–5	nd
Distance excretory pore to cephalic end	106–136	nd
Distance anus to caudal end	38–60	40
Oesophagus length (total)	207–395	209
Oesophagus length (cylindrical part)	137–145	131
Spicules length		
Shorter spicule	97–132	117^b^
Longer spicule	103–150	
Spicules maximum width	17–23	18
Gubernaculum length	39–54	48
Female	(*n* = 3)	
Body length (mm)	23–26	–
Body width	135–314	–
Cuticle thickness at mid-body	6–7	–
Distance excretory pore to cephalic end	132–222	–
Oesophagus length	267–273	–
Distance vulva to anus	94–105	–
Distance vulva to caudal end	176–184	–
Distance anus to caudal end	77–83	–
Larva	(*n* = 50)	–
Body length	310–408	–
Body maximum width	14–28	–

*Abbreviation*: *nd* not determined

^a^No females and larvae were measured from Germany

^b^The specimen was photographed from lateral view and only one spicule was visible

**Fig. 5:**
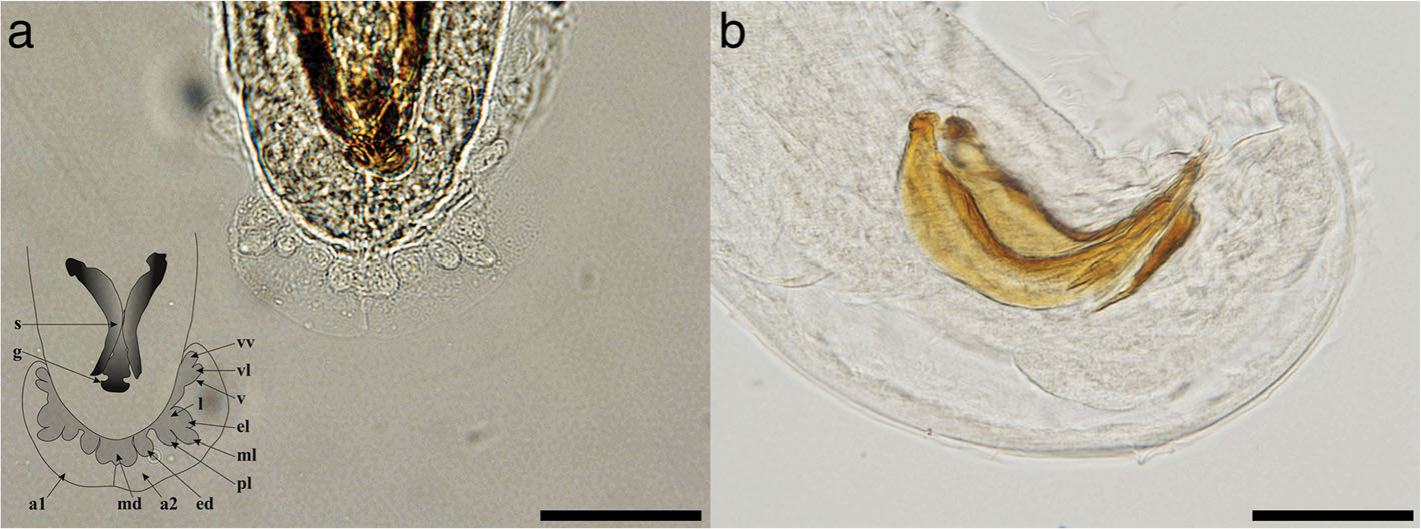
Light microscopy and schematic representation of the posterior end of the males of *Perostrongylus falciformis.* **a** Copulatory bursa, dorsal view (inset: schematic representation: a1, a2, lateral lobes; v, ventral ray; vv, ventro-ventral branch of ventral ray; vl, ventro-lateral branch of ventral ray; l, lateral ray; el, externo-lateral part of lateral ray; ml, medio-lateral part of lateral ray; pl, posterio-lateral branch of lateral ray; ed, externo-dorsal ray; md, median-dorsal ray; s, spicules; g, gubernaculum) **b** Copulatory bursa, lateral view. *Scale-bars*: 50 μm

**Fig. 6:**
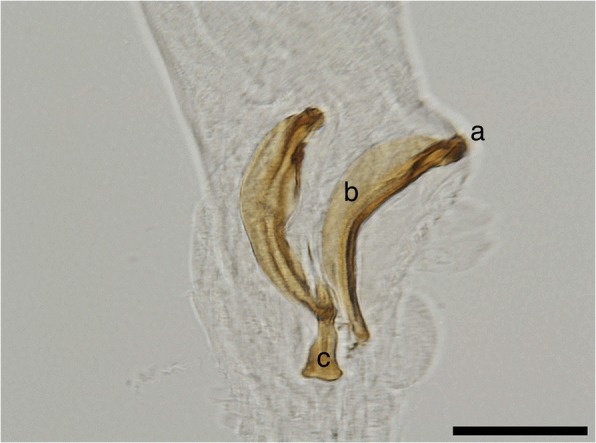
Spicules and gubernaculum of *P. falciformis*. **a** Knob-shaped anterior end of the spicule. **b** The bent caudal half of the spicule. **c** Gubernaculum. *Scale-bar*: 50 μm

The morphometric data for the males are shown in Table 3. At the posterior end, the males have a small copulatory bursa, two dissimilar and highly curved spicules, and a well-developed gubernaculum (Fig. 5). The copulatory bursa (Fig. 5a, b) is undeveloped, bi-lobed, with two symmetrical, transparent, and indistinguishable lateral lobes. The lobes are supported by rays with different appearance and origins: ventral, lateral, externo-dorsal and median. The ventral ray is short and distally split into two branches, the ventro-ventral and ventro-lateral, both similar in size. The lateral ray is divided into three short branches with lobate appearance: externo-lateral and medio-lateral having a common trunk, slightly separated from the postero-lateral branch. The externo-dorsal ray is undivided, small and lobated. The median-dorsal ray is short, thick, and has two lateral micro-lobes and a median, sharp and short expansion (Fig. 5a). The spicules are chitinous, brown, slightly dissimilar, sickle-shaped, short, but stout. The anterior end of each spicule is knob-shaped or hemispherical and is followed by a bent caudal half, sharpened on an edge and thickened on the opposite side (Fig. 6a, b). The gubernaculum is placed between the spicules, being attached to them through protractor and retractor muscles. It is triangular, with prolonged and tapered anterior half, while the posterior end is bifurcated with a bi-lobed shape at the base (Fig. 6c).

### Development of *P. falciformis* in *Cornu aspersum*

Larval stages of *P. falciformis* were found in 27 out of 30 (90 %) experimentally infected snails. Numbers and developmental stages of larvae detected from experimentally infected snails are shown in Table 4. All control *C. aspersum* specimens digested prior to the infection (*n* = 10) were negative for helminths.

**Table 4:** Total number (mean no. per snail ± SD) for the developmental stages of *Perostrongylus falciformis* larvae collected from five experimentally infected snails at 3 (T1), 6 (T2), 10 (T3), 15 (T4), 20 (T5) and 30 (T6) days post-infection

	First-stage larvae	Second-stage larvae	Third-stage larvae	Total
T1	19 (3.8 ± 2.7)	–	–	19 (6.3 ± 11.0)
T2	10 (2.0 ± 1.6)	–	–	10 (3.3 ± 5.8)
T3	13 (2.2 ± 1.8)	59 (11.8 ± 2.6)	1 (0.2 ± 0.4)	73 (24.3 ± 30.6)
T4	3 (0.6 ± 0.9)	78 (15.6 ± 7.2)	1 (0.2 ± 0.4)	82 (27.3 ± 43.9)
T5	4 (0.8 ± 1.1)	37 (7.4 ± 5.7)	4 (0.8 ± 1.1)	45 (15.0 ± 19.1)
T6	5 (1.0 ± 1.7)	36 (7.2 ± 3.8)	23 (4.6 ± 4.1)	64 (21.3 ± 15.6)

A total of 293 larvae were found at the gastropod digestions. First-stage larvae were found from T1 until the end of the study period, whereas the first L3 was detected as soon as 10 dpi and increasingly found until the end of the observational period (Table 4).

### Morphology of the larval stages of *P. falciformis*

All measurements below are given in micrometres (Table 5). Metrical data are given as the range, with the mean in parentheses.

**Table 5:** Measurements (in micrometres) of first- (L1), second- (L2) and third-stage (L3) larvae (*n* = 10 each) of *Perostrongylus falciformis*

Measurements	L1	L2	L3	Reference
Body length	247–352 (317 ± 41)	403–443 (421 ± 13)	459–496 (484 ± 12)	Present study
	270–370	350–420	340–440	[11]
	–	–	380–440	[63]
Maximum body width	13–18 (14 ± 1)	30–33 (31 ± 1)	23–33 (27 ± 3)	Present study
	10–17	26	30–32	[11]
	–	–	24-27	[63]
Tail length	23–37 (32 ± 5)	35–46 (41 ± 3)	30–43 (39 ± 4)	Present study
	33–40	–	30–46	[11]
Oesophagus length	99–163 (132 ± 25)	145–161 (153 ± 5)	162–196 (173 ± 10)	Present study
	130–150	161	130–183	[11]
Intestine length	130–150	204	176–260	[11]
Genital primordium, distance from posterior extremity	66–117 (88 ± 17)	–	109–166 (141 ± 15)	Present study
Nerve ring, distance from anterior extremity	–	–	66–76 (72 ± 3)	Present study
	85–90	–	–	[11]
Excretory pore, distance from anterior extremity	–	–	76–92 (84 ± 6)	Present study
	90–100	–	75–90	[11]
Ratio oesophagus length to body length	0.399–0.464	0.359–0.362	0.352–0.395	Present study
Ratio distance from posterior extremity to genital primordium to body length	0.267–0.331	–	0.237–0.334	Present study
Anus to posterior extremity	30	40	30	[11]

**Fig. 7:**
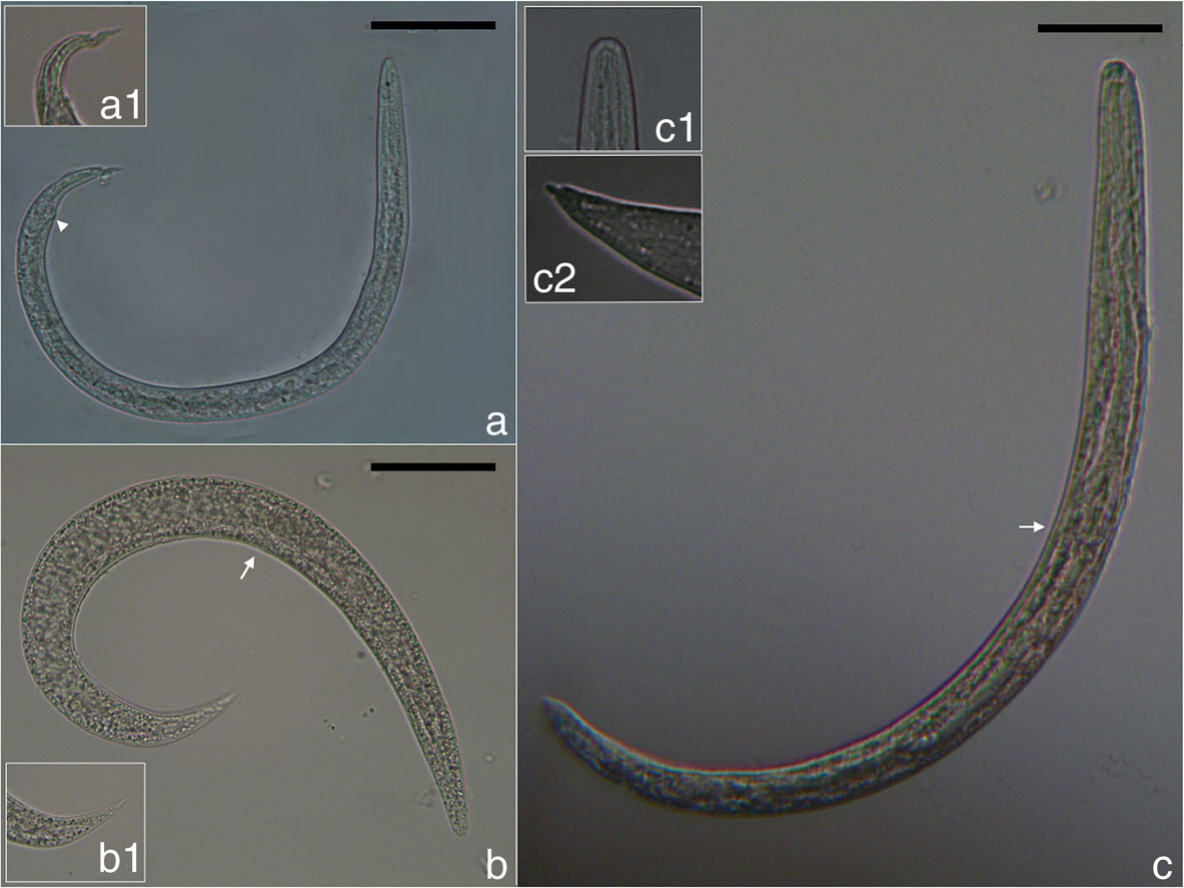
Larval stages of *P. falciformis*. **a** First-stage larva and detail of the posterior extremity (a1) and well-visible anus (arrowhead). **b** Second-stage larva and detail of the posterior extremity (b1) and oesophago-intestinal junction (arrow). **c** Third-stage larva and details of the anterior (c1) and posterior (c2) extremities and oesophago-intestinal junction (arrow). *Scale-bars*: 50 μm

First-stage larvae collected from European badgers (Fig. 7a) measured 247–352 (317 ± 41) in length and 13–18 (14 ± 1) in width. The anterior extremity was featured by a narrowed, blunt end with a terminal buccal opening. The posterior extremity was 23–37 (32 ± 5) in length and characterized by a dorsal subterminal spine with a deep notch and a ventral bulge followed by an elongated sigmoid ending (Fig. 7a1).

Second-stage larvae (Fig. 7b) measured 403–443 (421 ± 13) in length and 30–33 (31 ± 1) in width. L2 were C-shaped and filled with granules. The button-like anterior extremity as well the tail (Fig. 7b1) resembled that of L1. The anterior and posterior extremities displayed an empty-like appearance due to the presence of the cuticle of the L2 and the sheet of the previous larval stage.

Third-stage larvae (Fig. 7c) had a ventrally curved body measuring 459–496 (484 ± 12) in length and 23–33 (27 ± 3) in width. Some L3 were still encased in the sheets of the previous stages. The anterior end was blunt, with a distinct buccal cavity followed by two stylet-like structures (Fig. 7c1). The muscular upper part of the oesophagus was cylindrical and followed by the glandular part which gradually enlarged in the bulbar oesophago-intestinal junction. The nerve-ring and the slightly posterior excretory pore were detected at 66–76 (72 ± 3) and 76–92 (84 ± 6) from the anterior extremity, respectively. The posterior extremity was 30–43 (39 ± 4) in length with a digitiform tip (Fig. 7c2).

### Molecular data and phylogenetic position of *P. falciformis*

For molecular analysis of *P. falciformis* a region of 4021 bp of the ribosomal DNA including the near complete *18S*, ITS1, *5.8S*, ITS2 and partial *28S* and a partial region of 1075 bp of the mitochondrial cytochrome *c* oxidase subunit 1 gene (*cox*1) were sequenced from the German isolates. Furthermore, in order to compare geographical variants, the ITS2 region for *P. falciformis* isolates from Bosnia and Herzegovina and the ITS2 region and a partial *cox*1 gene for isolates from Romania were sequenced. The *cox*1 sequence from Romania was 99% identical to the German isolate, leading to three residue changes in the deduced amino acid sequence. The obtained ITS2 sequences of *P. falciformis* from the three countries were 100% identical, except one clone, suggesting an overall low geographical variation. The one exceptional rDNA clone (GenBank: KY365436) of the amplicon NF1-NC2 was from a female fragment from Germany, which had 15 SNPs (1802/1817 bp identities) compared to sequences from six other clones. This could be an additional haplotype or a rare intraspecific sequence variation in the rDNA repeats.

GenBank database searches with the *P. falciformis 18S* and *28S* rDNA sequences did not support the close phylogenetic relationship to *A. abstrusus* as would have been expected from the current taxonomic classification where *Perostrongylus* is considered as a subgenus of *Aelurostrongylus* [17]. Among the best matches for *P. falciformis*, according to alignment scores were species of the genera *Parafilaroides* and *Filaroides*. In contrast, the alignment score for *A. abstrusus* was lower and in the same range than to other metastrongyloid genera (not shown).

**Fig. 8:**
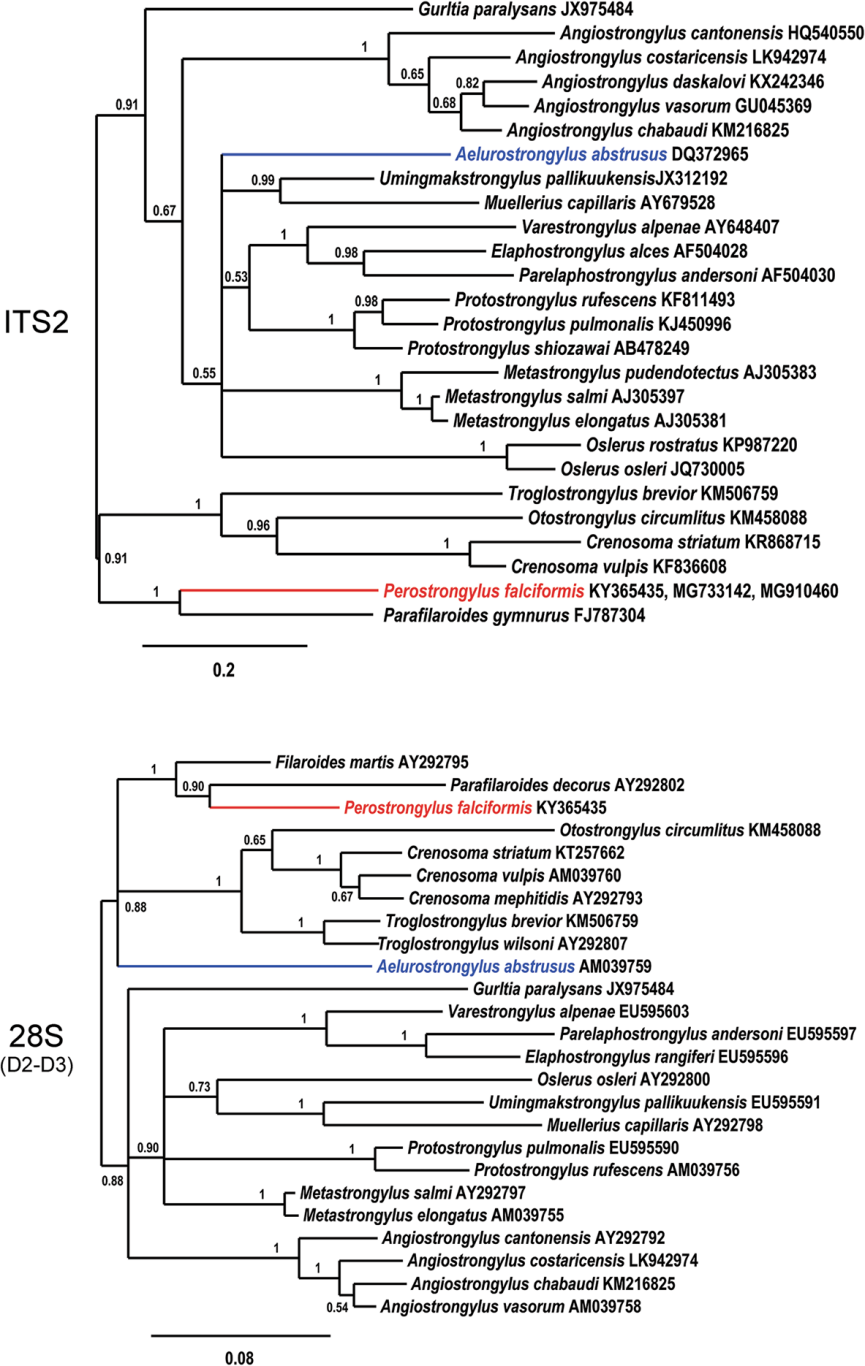
Phylogenetic relationships of *P. falciformis* (red) with *A. abstrusus* (blue) and other metastrongyloid nematodes. Phylogenetic analysis based on ITS2 and partial *28S* rDNA (domains D2-D3) sequences of metastrongyloid genera. Trees are constructed using Bayesian inference. Posterior probability values are shown next to the nodes; branches with values < 0.5 are not shown. The scale-bar indicates the number of substitutions per site

**Fig. 9:**
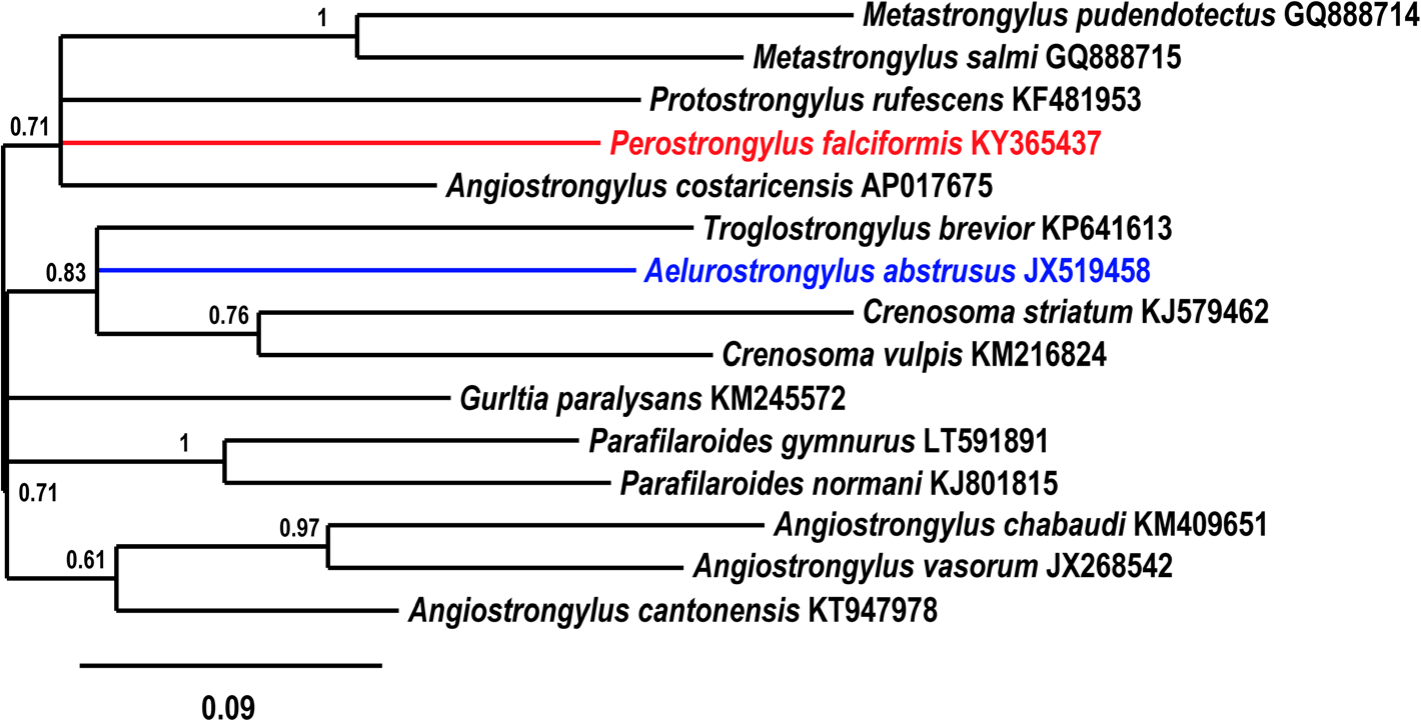
Phylogenetic tree (Bayesian inference) using *cox*1 sequences for *P. falciformis* (red), *A. abstrusus* (blue) and species of other metastrongyloid genera. Posterior probability values are shown next to the nodes; branches with values < 0.5 are not shown. The scale-bar indicates the number of substitutions per site

To further investigate the relationships among *Perostrongylus* and other metastrongyloid nematodes and to determine the molecular phylogenetic relation, analyses were performed with ITS2, partial *28S* (domains D2-D3) (Fig. 8) and partial *cox*1 sequences (Fig. 9) as biomarkers. These sequences were chosen due to their higher resolution at the species level and their length was adjusted to include a maximum of high scoring metastrongyloid nematodes from BLAST search on GenBank.

In the phylogenetic trees, *P. falciformis* and *A. abstrusus* were clearly separated, assigned to different clades, which was supported by all three genetic markers. Interestingly and consistent with discussed morphological similarities and forms of reproduction, the phylogeny inferred from the two rDNA sequence analyses, grouped *P. falciformis* together with species of the genera *Parafilaroides* and *Filaroides* (both within the family Filaroididae Schulz, 1951). The phylogenetic relationships of the Metastrongyloidea rDNA sequences correspond to previous studies [2]. There was strong support for groups of congeneric species and for the exclusion of the family Crenosomatidae from other metastrongyloid taxa. The relations obtained here for the partial *cox*1 sequence seem less consistent, because some morphologically proven congeneric species grouped with unrelated families, e.g. *A. abstrusus* with the Crenosomatidae and *A. costaricensis* with the Metastrongylidae.

### Pathology caused by *P. falciformis* in European badgers

**Fig. 10:**
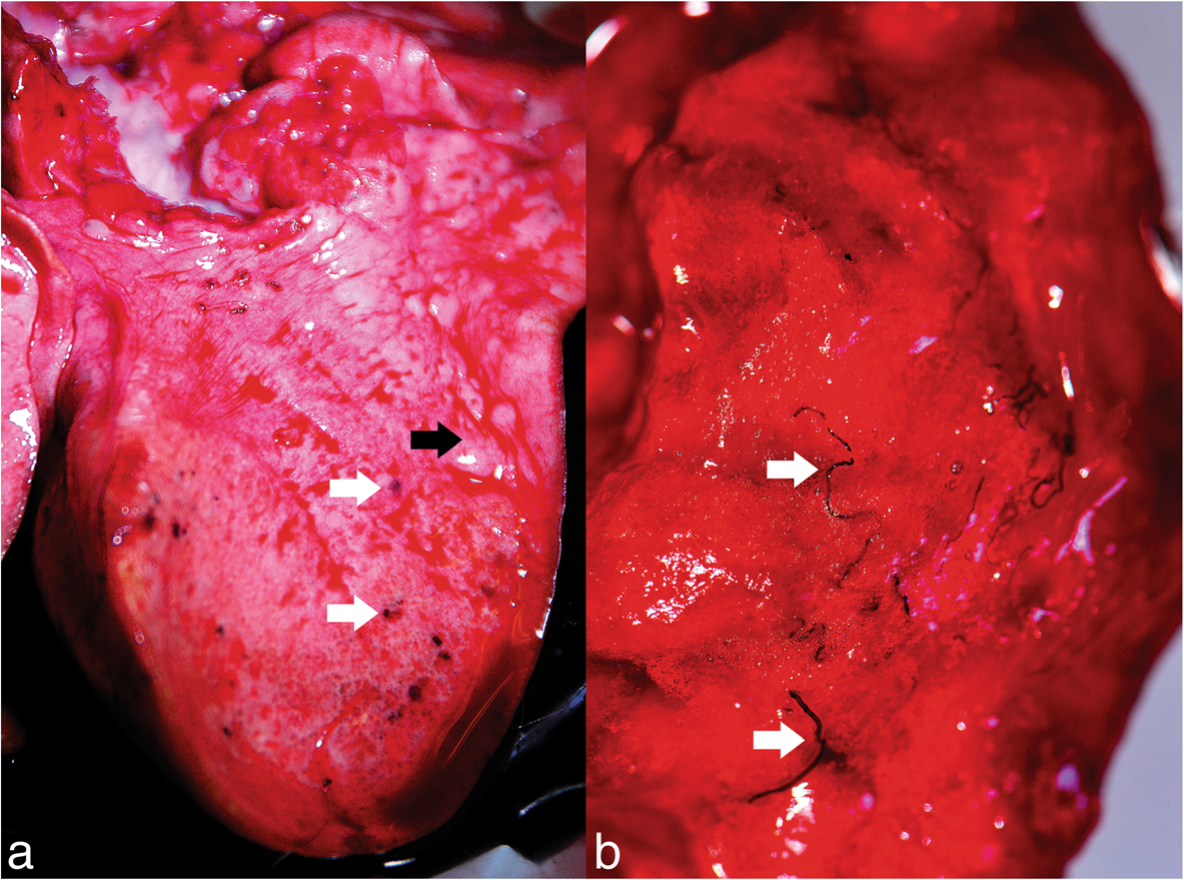
Gross lesions produced by *P. falciformis*. **a** Adults in nodules in the subpleural space (white arrows) and the presence of alveolar emphysema (black arrow). **b** Adults in the pulmonary parenchyma (white arrows)

Grossly, multifocal, slightly elevated, well defined, small brown-black nodules were randomly distributed in the subpleural region of both lungs (Fig. 10a). Frequently, the parasite-containing nodules were associated with multifocal to coalescing areas of moderate alveolar emphysema (Fig. 10a). On the cut section, multiple, equally thin, blackish, partially coiled, *P. falciformis* adults were embedded in the lung parenchyma (Fig. 10b), without a significant preference for certain lung lobes. The bronchi were filled with a mucinous, foamy exudate (Fig. 10a).

**Fig. 11:**
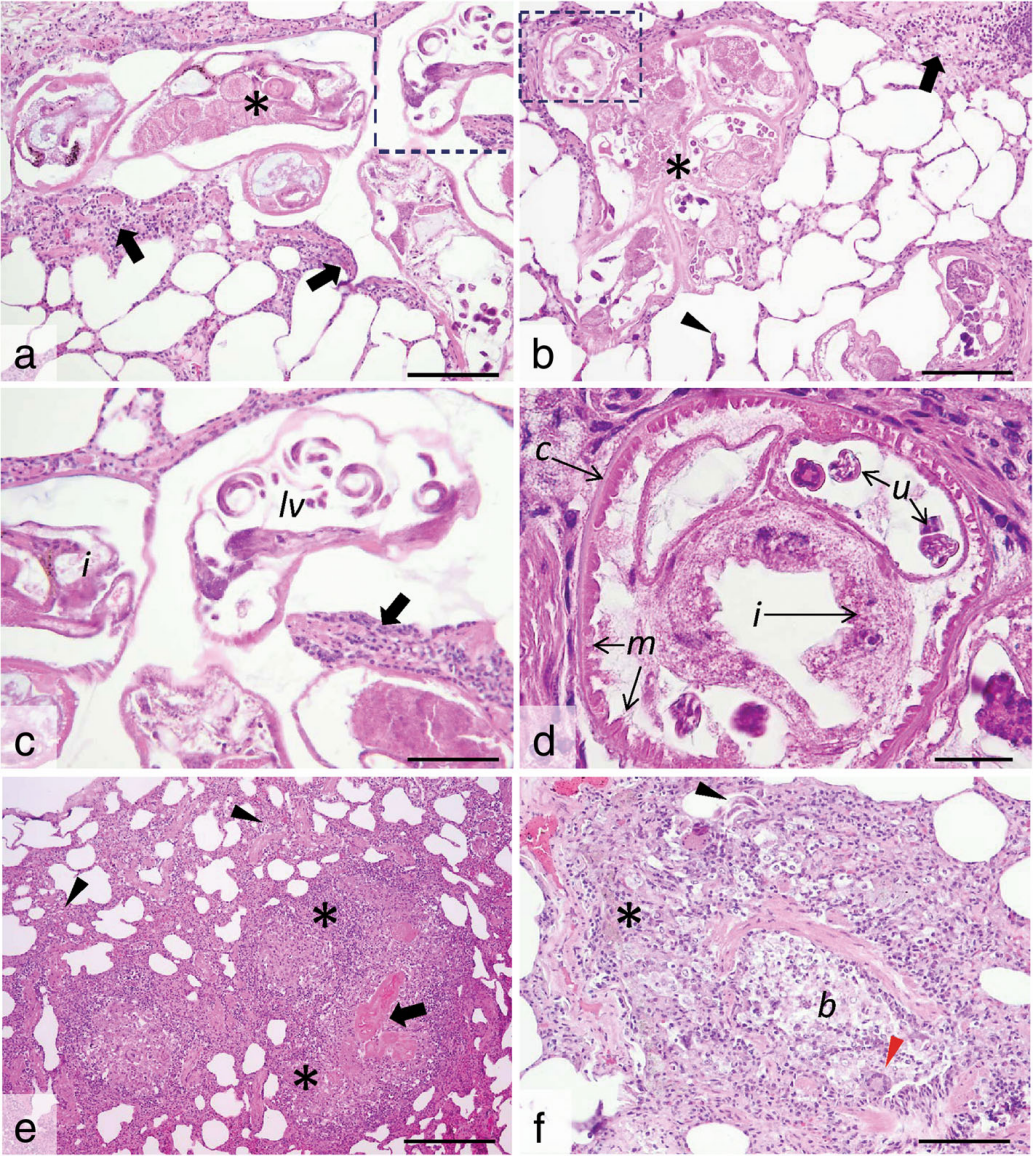
Histological cross- and tangential sections of *P. falciformis* in the lung parenchyma. **a**, **b**. Viable *P. falciformis* adults (asterisks) coiled in the lung parenchyma and surrounded by a mild leukocyte reaction (many macrophages, occasional multinucleate giant cells, some lymphocytes and eosinophils) (black arrows) and smooth muscle hyperplasia. The alveolar walls are moderately expanded by the above described inflammatory cell population, with occasional alveolar wall rupture (arrowhead) and emphysema. **c**, **d** Detail of the marked areas in **a** and **b**. The parasites have a thin smooth cuticle (*c*) and pseudocoelom, coelomyarian-polymyarian musculature (*m*), intestine (*i*) with granular pigment and large uterus (*u*) filled with developing larvae (*lv*). The leukocyte reaction is also visible (black arrow in **c**). **e**, **f**. Free *P. falciformis* larvae (with thin walls and granular content) (black arrowhead in **f**) are present in the bronchiolar (*b*) and peribronchiolar spaces, associated with prominent inflammatory reaction consisting of many macrophage (some laden with hemosiderin) (asterisk in **f**), epithelioid cells and multinucleate giant cells (red arrowhead in **f**) and few eosinophils and fibroblast. Degenerated parasites (black arrow in **e**) induce a marked inflammatory response consisting of ill-defined granulomas (asterisks in **e**) and a locally-extensive interstitial lympho-histiocytic and eosinophilic pneumonia (arrowheads in **e**); H&E staining, ×20 (**a**, **b** and **f**), ×40 (**c**), ×100 (**d**) and ×10 (**e**). *Scale-bars*: **a**, **b**, **f**, 100 μm; **c**, 50 μm; **d**, 25 μm; **e**, 200 μm

Histologically, *P. falciformis* adults embedded in the lung parenchyma were present in high number in all examined histological sections. Few larval stages were also noted, especially in the bronchioles. *Perostrongylus falciformis* adults have a thin smooth cuticle, coelomyarian-polymyarian musculature and pseudocoelom with prominent intestine lined by cells that frequently contain brown-black granular pigment, and large uterus filled with developing eggs and larvae (Fig. 11c). Viable *P. falciformis* adults induce a mild inflammatory response consisting of macrophages (frequently laden with hemosiderin), occasional multinucleate giant cells, some lymphocytes and eosinophils (Fig. 11a-c). Few fibroblasts and thin collagen bundles were scattered between the above described cells. Smooth muscle hyperplasia of the terminal airways and alveolar emphysema were occasionally associated with the foci of interstitial pneumonia. Some viable adult nematodes are directly surrounded by a thin fibrous capsule. The inflammatory infiltrate extends from the parasite to the adjacent parenchyma, markedly expanding the alveolar walls. Alveolar emphysema was also focally associated with the foci of interstitial pneumonia. Bronchus-associated lymphoid tissue hyperplasia and alveoli filled by cellular infiltrate (as described above) and oedema were also observed.

Degenerated adult parasites (Fig. 11e) induced a marked inflammatory response consisting of poorly localized granulomas (sometimes centred on the parasite debris) and lympho-histiocytic and eosinophilic interstitial pneumonia. Occasionally, free *P. falciformis* larvae were present in the peribronchiolar and bronchiolar spaces (Fig. 11f). At these sites, the bronchiole walls were segmentally infiltrated by macrophages, epithelioid cells and multinucleate giant cells admixed with few eosinophils, fibroblasts and coiled larvae. Additionally, moderate to severe smooth muscle and bronchiolar epithelial hyperplasia with luminal obstruction by sloughed epithelial cells admixed with mucus, leukocytes (as described above) and parasite larvae were noted.

## Discussion

This study provides the first molecular evidence for the validity of the genus *Perostrongylus*, which was previously synonymized with or considered a subgenus of *Aelurostrongylus*. With the exception of *A. abstrusus*, other species previously assigned to genus *Aelurostrongylus*, namely *A. brauni* and *A. fengi* were subsequently transferred to different genera. Due to the extremely poor description of *A. brauni* (a parasite originally described as *Strongylus brauni* from the Indian civet, *Viverra zibetha*), its classification as *species inquirenda* has already been suggested [19] and is now designated as *Viverrostrongylus brauni* [20]. *Aelurostrongylus fengi* was described from Crab-eating mongoose, *Herpestes urva*, as *Pulmostrongylus fengi*. The taxonomic status of *Pulmostrongylus* (which includes already several species) is under debate, and currently it is considered a valid genus [18] or a subgenus of *Protostrongylus* [16]. Our molecular data provide evidence that *P. falciformis* and *A. abstrusus* are not congeneric. The conclusion drawn from this is the validity of the full genus status of *Perostrongylus* and the species name *P. falciformis* for the European badger lungworm.

Hence, the genus *Aelurostrongylus* becomes monotypic and includes only the type-species *A. abstrusus*. Considering the rigorous rules of taxonomy, an interesting fact arises regarding *A. abstrusus*. As the first description of the species used the specific name *pusillus* [4], the valid name for this species should be *Aelurostrongylus pusillus* (see above). However, due its veterinary importance and worldwide use since more than a century under the name *A. abstrusus*, we suggest the latter name to be used.

From a phylogenetic standpoint, *Perostrongylus* is a valid genus and most probably includes two species: *P. falciformis*, a parasite of *M. meles* and possibly *M. erminea* in Europe, and *P. pridhami* [21], a parasite of *N. vison* and *Mustela erminea* in North America. Our view is strongly supported by molecular phylogenetic data currently available only for *P. falciformis*. Further molecular studies are needed also for *P. pridhami* in North America, to conclude its phylogenetic position and relationships to *P. falciformis*.

Moreover, the phylogenetic analysis of the relationships of *P. falciformis* with species of other metastrongyloid genera clearly assigned *Perostrongylus* and *Aelurostrongylus* to different clades. *Perostrongylus falciformis* was grouped with high support together with the genera *Filaroides* and *Parafilaroides* (family Filaroididae), whereas *A. abstrusus* represented a single branch with no sister groups. Alignments of the internal transcribed spacer sequences (ITS1, ITS2) for different genera of the Metastrongyloidea are difficult because of the high variability in comparison to ribosomal RNA genes (*18S*, *28S*). We therefore performed the ITS2 multiple sequence alignment using a method of the program MAFFT which also considers secondary structures. However, the obtained correspondence between the analyses of ITS2 and the *28S* rDNA D2-D3 region corroborate the inferred phylogenetic position of *P. falciformis* close to the two genera of the Filaroididae within the superfamily Metastrongyloidea.

The close relationship of *Perostrongylus*, *Filaroides* and *Parafilaroides* in the molecular phylogeny is in good agreement with morphological characters and forms of reproduction. Males of species in all three genera have short, stout and arcuate spicules, a single-element gubernaculum and females are ovoviviparous. In contrast, males of *A. abstrusus* have slender (half the width of *P. falciformis*) and straight spicules, a gubernaculum of two joined equal elements and the females are oviparous [55]. The present paper also provides a very detailed morphological description of the adult stages of *P. falciformis* which was previously relatively brief [8, 10].

Both species of genus *Perostrongylus* are parasites of the Mustelidae. *Perostrongylus falciformis* has been reported so far only in European badgers from various countries in the western Palaearctic, including Germany [8, 11], Ukraine [56], Russia [57], Italy [58], UK [26], Norway [27], Poland [28], and Bosnia and Herzegovina [29]. Here we report for the first time the presence of *P. falciformis* in Romania. In a study from UK on stoats, unidentified nematodes were shown in a histological section of a lung [59]. Later, Simpson et al. [60], hypothesized, based on the morphology of the female in these histological sections that the nematodes could be *P. falciformis*. However, without morphological or genetic data, this remains only a presumption and we do not list stoats as confirmed hosts for *P. falciformis*.

*Perostrongylus pridhami* has been reported mainly in the American mink, *N. vison* in North America: Ontario, Canada [21, 22, 37, 42], Newfoundland, Canada [30], and Montana, USA [40] and in stoats, *M. erminea* from Newfoundland [30]. Torres et al. [32] mentioned *P. pridhami* in European badgers in Spain. However, this is highly unlikely, as it probably represents a misidentification of *P. falciformis*.

Interestingly, one study on invasive American minks in Spain [33] reported a specimen identified as *Aelurostrongylus* spp. As the specimen was not identified to the species level, it is not known if this represents *P. pridhami*, a natural parasite of this host in North America, but in an invasive population, or *P. falciformis*, which could suggest an adaptation from European badgers to invasive American minks.

Lungworms of genus *Perostrongylus* seem to occur with variable prevalence in mustelids across their distribution range (Table 6). Additionally to the overview in this table, unidentified lungworms were reported in 18% (8/45) of European badgers in Germany, with pathological lesions consistent with those produced by *P. falciformis* [61]. In our opinion, a low prevalence or total absence of these parasites in studies with a reasonably large number of samples are likely the result of a rather superficial examination; probably *P. falciformis* is present in European badgers across their distribution range.

**Table 6:** Reported prevalence for *Perostrongylus* spp.

Species	Host	Country	Prevalence (%) (infected/examined)	Reference
*P. falciformis*	*Meles meles*	Germany	50.0 (6/12)	[10]
*P. falciformis*	*Meles meles*	Poland	22.2 (2/9)	[28]
*P. falciformis*	*Meles meles*	Norway	33.3 (3/9)	[27]
*P. falciformis*	*Meles meles*	UK	0.8 (1/118)	[26]
*P. falciformis*	*Meles meles*	Italy	52.6 (10/19)	[58]
*P. falciformis*	*Meles meles*	Spain	3.5 (3/85)	[32]
*P. falciformis*	*Mustela erminea*	UK	13.5 (5/37)	[59]
*P. pridhami*	*Neovison vison*	Canada	8.6 (13/152)	[42]
*P. pridhami*	*Neovison vison*	Canada	2.1 (1/48)	[30]
*P. pridhami*	*Mustela erminea*	Canada	12.5 (8/40)	[30]
*Perostrongylus* sp.	*Neovison vison*	Spain	2.0 (1/50)	[33]

The data presented here indicate that *C. aspersum* is a suitable intermediate host of *P. falciformis*. However, in a series of laboratory infection studies, larval development from L1 to L3 was demonstrated to occur in two slug species [*Deroceras agreste* (Linnaeus, 1758) and *Arion hortensis* (Férussac, 1819)] and five species of terrestrial snails [*Trochulus hispidus* (Draparnaud, 1801), *Cepaea hortensis* (Müller, 1774), *C. nemoralis* (Linnaeus, 1758), *Euomphalia strigella* (Draparnaud, 1801) and *Succinea putris* (Linnaeus, 1758)] [11, 62].

After 24 hours, the L1 were found coiled between the muscle fibres of the snails’ foot and the first moult occurred at 6–8 dpi at room temperature (or as long as 14 days at lower temperatures) [11, 62]. In the present study, the first L2 larvae were found at 10 dpi (however, the previous examination time was 6 dpi). Wetzel [11] mentions the second moult at 10–12 dpi. In our study, the first L3 were found at 10 dpi, but the largest numbers were present in snails at 30 dpi. Wetzel [11] also mentioned that these time frames are slightly variable according to the species of snail used, but no other details were provided. Wetzel [11, 62] described in detail L1-L3 larval stages and provided drawings for L1 and L3. These morphological details are largely consistent with our results (Table 5), with only minor differences. In addition, our present work provides the first detailed photomicrographs of L1-L3.

The life-cycle of *P. pridhami* described in North America [21] showed a role as potential intermediate hosts for several experimentally infected species of aquatic snails [*Physa integra* (Haldeman, 1841), *Gyraulus deflexus* (Sandberger, 1858), *G. crista* (Linnaeus, 1758), *Ampullaria cuprina* Reeve, 1856] as well as terrestrial snails [*Zonitoides arboreus* (Say, 1816), *Discus cronkhitei* (Newcomb, 1865), *Novisuccinea ovalis* (Say, 1817), *Anguispira alternate* (Say, 1816)] or slugs [*Deroceras gracile* (Müller, 1774)] [21]. As in *P. falciformis* [62], larvae of *P. pridhami* penetrate the foot of snails [21]. Stockdale [37] succeeded to infect terrestrial snails by injection. The dynamics of larval development in snails is not known for *P. pridhami*. The prepatent period for this species was 23–28 days in experimentally infected minks [21], slightly longer than for *P. falciformis* in European badgers [11, 62]. As demonstrated for other lungworms of carnivores, *P. pridhami* can be transmitted to minks after ingestion of paratenic hosts (mice, birds, amphibians and fish) [21] but no such information is available for *P. falciformis*.

Additional information is provided on the development of *P. pridhami* in minks experimentally infected *via* gastric tube with L3 from terrestrial snails [37]. The first moult in minks (L3 to L4) occurred 3 dpi and the final moult (L4 to L5) at 7 dpi [37]. The migration in the minks included penetration of the stomach wall, crossing the peritoneal cavity and the diaphragm, followed by penetration of the visceral pleura of the lungs, all these occurring within the first 24 hours [37]. The migration pattern and last two moults for *P. falciformis* in European badgers are not known. Anderson [21] also described the morphology of L1-L3 of *P. pridhami* which seem to be similar to that of the larvae of *P. falciformis* ([11, 62]; present study). L1-L3 of *P. pridhami* are all illustrated in detail [21].

As concluded by Hancox [25], although the European badger is affected by a wide range of parasites, average rates of infection will not have a significant effect on host population regulation. However, individuals may be affected by a high parasite load. Lesions and symptoms produced by *P. falciformis* in European badgers have been described previously [8, 10, 27, 29, 60]. Additionally, lesions consistent with a possible *P. falciformis* infection in *M. erminea* were found in the UK [59, 60]. In North America, lesions produced by *P. pridhami* in minks have also been described [21, 37, 38]. Generally, the pathology caused by the two species of *Perostrongylus* is similar and results in vomiting (though after experimental infection), coughing (usually when large numbers of parasites are present) and can be complicated by the presence of co-infections [i.e. with *Filaroides martis* (Werner, 1782) in minks]. However, the clinical signs are known only from experimental infections. The importance of co-infections with *Angiostrongylus daskalovi* Janchev & Genov, 1988 in European badgers [63] on the clinical outcome is unknown.

The lesions are produced either by infective larval migration and are located at various levels (stomach, peritoneum, pleura) or by larvae migrating through the lung parenchyma and adults. Generally, adults of *P. falciformis* are found either in subpleural granulomas or embedded in the lung parenchyma (present study). Similar subpleural nodules were found also in minks infected with *P. pridhami* [37, 38]. The histological lesions in our study are to date the most detailed and largely similar to the lesions observed in other studies on *P. falciformis* [27, 29, 59] or *P. pridhami* [38].

## Conclusions

Molecular phylogenetic and morphological data support the validity of the genus *Perostrongylus*, with probably two species: *P. falciformis* in European badgers in Europe and *P. pridhami* in minks in North America. Moreover, *Aelurostrongylus* becomes a monotypic genus, with *A. abstrusus* as the type- and only species. Interestingly, the two genera clearly belong to two different evolutionary branches: *Perostrongylus* is grouped together with the genera *Filaroides* and *Parafilaroides* (both in family Filaroididae), whereas *Aelurostrongylus* belongs to a single branch, with no sister groups. The present study also demonstrated for the first time that *C. aspersum* snails are suitable intermediate hosts. Several aspects remain unknown. One question is which species of *Perostrongylus* is found in American minks invasive to Europe. The role of paratenic hosts in the life-cycle of *P. falciformis* is also a matter to be explored. Moreover, molecular data from *P. pridhami* will bring further proof for the phylogenetic position of *Perostrongylus* among metastrongyloids.
